# Synthesis of Prostate MR Images for Classification Using Capsule Network-Based GAN Model

**DOI:** 10.3390/s20205736

**Published:** 2020-10-09

**Authors:** Houqiang Yu, Xuming Zhang

**Affiliations:** 1 Ministry of Education Key Laboratory of Molecular Biophysics, Department of Biomedical Engineering, School of Life Science and Technology, Huazhong University of Science and Technology, No 1037, Luoyu Road, Wuhan 430074, China; yuhouqiang@hbust.edu.cn; 2 Department of Mathematics and Statistics, Hubei University of Science and Technology, No 88, Xianning Road, Xianning 437000, China

**Keywords:** prostate cancer, MR image, image synthesis, GAN, capsule network

## Abstract

Prostate cancer remains a major health concern among elderly men. Deep learning is a state-of-the-art technique for MR image-based prostate cancer diagnosis, but one of major bottlenecks is the severe lack of annotated MR images. The traditional and Generative Adversarial Network (GAN)-based data augmentation methods cannot ensure the quality and the diversity of generated training samples. In this paper, we have proposed a novel GAN model for synthesis of MR images by utilizing its powerful ability in modeling the complex data distributions. The proposed model is designed based on the architecture of deep convolutional GAN. To learn the more equivariant representation of images that is robust to the changes in the pose and spatial relationship of objects in the images, the capsule network is applied to replace CNN used in the discriminator of regular GAN. Meanwhile, the least squares loss has been adopted for both the generator and discriminator in the proposed GAN to address the vanishing gradient problem of sigmoid cross entropy loss function in regular GAN. Extensive experiments are conducted on the simulated and real MR images. The results demonstrate that the proposed capsule network-based GAN model can generate more realistic and higher quality MR images than the compared GANs. The quantitative comparisons show that among all evaluated models, the proposed GAN generally achieves the smallest Kullback–Leibler divergence values for image generation task and provides the best classification performance when it is introduced into the deep learning method for image classification task.

## 1. Introduction

Prostate cancer is a malignant tumor that will pose great threats to middle-aged and elderly males. According to the Global Cancer Report 2018 issued by the International Agency for Research on Cancer (IARC) of the World Health Organization, the incidence rate of prostate cancer ranks the second and its mortality rate is in the top five among male tumors in the world [1]. Early diagnosis is critical for reducing the harm of prostate cancer. Magnetic resonance imaging (MRI) has become the optimal imaging technique for the detection and diagnosis of prostate cancer due to its higher accuracy over transrectal ultrasonography (TRUS) and prostate-specific antigen (PSA) [2]. With the development of MRI technology in recent years, multiparametric MRI (mp-MRI), e.g., T2-weighted imaging (T2W), dynamic contrast-enhanced imaging (DCE), and apparent diffusion coefficient (ADC) images, can clearly display the anatomical and functional information of prostate regions due to different information from various modalities [3]. Compared with the single modality, mp-MRI has shown better diagnostic ability. Therefore, it has become an important technology for prostate cancer diagnosis [4].

In clinic, the diagnosis and treatment of prostate cancer based on MR images usually require experienced radiologists to interpret a large number of MR images, which is quite time consuming. In addition, the clinical diagnosis can be affected by significant variability from physicians [2]. In order to reduce the work burden of clinicians and the variability among observers, the Computer-Aided Diagnosis (CAD) system has aroused much interest. In the traditional CAD methods, the low-level features or handcrafted features are firstly extracted and then these features are utilized to represent images and train separate classifiers. Although this strategy can be a great success for certain data and tasks, the design of effective feature extractors for new tasks and data requires new domain knowledge and experience because most handcrafted features cannot simply be adapted to new conditions [5,6]. Another category of CAD methods is to utilize the deep learning approach because it needs less manual intervention and can automatically extract the intrinsic features from the original data [7]. It has become the state-of-the-art CAD technology. However, a major limiting factor is that the training of deep learning models depends on vast amounts of annotated medical data [8]. The collection and labeling of medical image data are costly, tedious and time consuming, which may lead to a severe lack of labeled training images. In addition, many datasets suffer from serious class imbalances in the medical imaging field due to the rarity of some pathologies. 

To address the problem of the lack in annotated medical images, the common approach is to augment data by applying the rigid or non-rigid transformations on the limited medical images. Although the number of samples can be increased in this way, it cannot ensure that the diversity of samples can be substantially improved. Therefore, this method still cannot address the possible over-fitting problem in training the deep learning models [9]. Another strategy of data augmentation is to synthesize “unseen” medical images with the aim of increasing the quantity of data and addressing the problem of lack in diversity of data. The typical example is the Generative Adversarial Networks (GAN) introduced by Goodfellow et al. [10]. Recently, many GAN-based models and their extensions have facilitated tackling the challenging image analysis problems such as segmentation [11], denoising [12], registration [13], detection [14] and classification [15]. As regards deep learning-based image classification, GAN has been widely for synthesizing training samples because its unique ability in mimicking data distributions indeed opens the possibility to bridge the gap between learning data and synthetic data [8]. GAN has achieved the great success and outperformed the traditional methods in data generation. The reasons for the superiority of GAN are related to two basic properties. Firstly, as an unsupervised training method, GAN aims to obtain useful information from data through an indirect way of supervision [16]. Secondly, GAN has shown significant performance improvement for the extraction of visual features by discovering the high-dimensional hidden statistical regularities of data [17].

A typical GAN consists of a generator and a discriminator. The two modules play an adversarial game, in which the generator is utilized to generate the fake data based on the learned distribution of real data and simultaneously the discriminator acts as a critic to determine whether the data it has received is from the real data distribution or the generator’s output distribution. The existing GAN-based models are different from each other in the design of the two modules and loss functions. Deep Convolutional GAN (DCGAN) has been proposed by Radford et al. [18] to address the instability drawback of the basic GAN model by using the deep convolutional network architecture in both the generator and the discriminator instead of the original multilayer perceptrons. This network shows highly competitive performance in an unsupervised learning fashion to extract hierarchical features of the image. To be specific, batch normalization and leaky-ReLU, as the critical components of the DCGAN, are utilized to enhance the stability of the model. Despite the advantage of DCGAN over the original GAN, mode collapse is prone to occur. The Wasserstein-GAN (WGAN) is another state-of-the-art model [19,20], which uses the Wasserstein-1 distance or the Earth Mover (ME) distance to replace the original Jensen-Shannon (JS) divergence of GAN. The WGAN model follows a common DCGAN architecture both on the discriminator and the generator. Through a more robust learning process, the WGAN provides a stable adversarial image generation model, which can discover deeper relationships between distributions. Despite these theoretical advantages, a slow optimization process is caused by the WGAN in actual scenarios. In order to solve the instability of GAN, another approach named Least Squares GAN (LSGAN) is proposed by Mao et al. [21]. In the LSGAN, the least squares loss is adopted for both the discriminator and the generator to avoid the vanishing gradient problem. In this way, the fake sample that is discriminated to be true but far away from the dense distribution of real samples will be penalized due to its distance from their main mode. Additionally, the gradient will become zero only when the distribution of real samples is perfectly matched with that of the fake sample. As a result, the LSGAN can perform more stable during the learning process and generate higher quality images over the regular GANs.

Although GANs and their extensions have achieved great success in the field of computer vision, medical images are more complex than natural images [22] because they generally contain plenty of important lesion information for the detection and diagnosis of certain kinds of diseases such as tumors or tuberculosis. The fine-grained objects are the main focus in the medical images. However, the size, shape and spatial relationship among various targets will be changed in different environments and acquisition conditions, which can affect the pose features of imaging objects. The traditional convolutional neural networks (CNN)-based architectures lack the ability to interpret complicated translational or rotational relationships [23], and they only need to detect the existence of features in the image. Motivated by the working mechanism of optic neurons in the human visual system, Sabour et al. [24] have introduced the capsule network as a powerful alternative to CNN. To address the loss of information caused by CNN, the capsule network learns a more equivariant image representation that is robust to the changes in the pose and spatial relationship of objects in the images. The training algorithm of the capsule network involves a routing mechanism between capsules in its successive layers that imitates the communication between human neurons which are responsible for visual perception and understanding. The initial intuition for designing deep neural networks is to model the hierarchical feature recognition from low-level attributes towards complex entities by imitating the learning process of human brains. Capsules are more effective than CNNs in capturing this intuition due to the in-built designed mechanism. The experiments on MNIST digit classification and segmentation of overlapping digits have showed that the capsules outperform the CNN-based state-of-the-art methods.

Inspired by the success of capsules, we have proposed to replace CNNs with the capsule architecture and incorporated the latter into GAN to improve its performance. Meanwhile, we have adopted the least squares loss as an alternative to the sigmoid cross entropy loss in the proposed capsule network-based GAN (CapGAN) model to overcome the vanishing gradient problem. Extensive experiments on the simulated MR images and the real prostate MR images demonstrate that the images generated by our model look more realistic and have higher quality than those produced by the compared GANs. The quantitative comparisons show that among all the evaluated GANs, our GAN achieves the best Kullback–Leibler (KL) divergence values in most cases. Furthermore, we have introduced the proposed image generation technique into prostate MR image classification method based on Laplacian eigenmaps network (LENet) [25] and network-in-network (NIN) [26]. It is demonstrated that the proposed CapGAN can help the combined LENet and NIN to classify prostate MR images with higher accuracy than other data augmentation methods. 

The remainder of the paper is organized as follows. Section 2 provides a brief description of GANs and the capsule network. Section 3 presents the proposed CapGAN model, and some details of its implementation. In Section 4, extensive experiments are conducted to evaluate CapGAN in terms of generated image quality, classification accuracy and complexity. Finally, several concluding remarks are given, and some future research directions are discussed. 

## 2. Preliminaries 

### 2.1. Generative Adversarial Network

**Figure 1:**
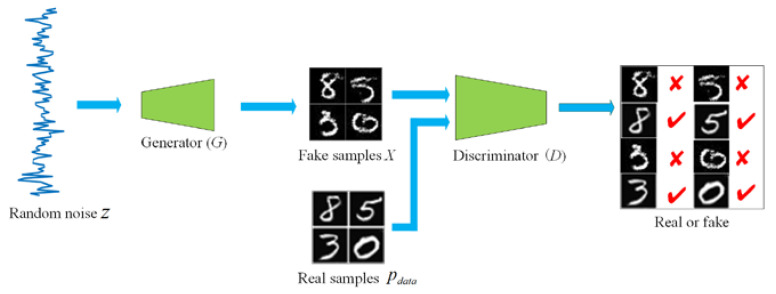
The architecture of generative adversarial network.

GAN is composed of a generator and a discriminator. The generator is used to generate the fake samples $X\in p_{fake}$, where the distribution of X is nearest to the real data distribution $p_{data}$. The discriminator plays the role in determining whether its input is from the real data distribution $p_{data}$ or the fake distribution $p_{fake}$ generated by the generator. GAN architecture is described in Figure 1, in which a uniform noise distribution Z as the initial input is followed by a generator *G* to up-sample it up to an image. The discriminator *D* will distinguish the fake samples from the real ones. The output of *D* is a probability score between 0 and 1 which represents the degree that the input image is real or fake. As the generator keeps learning, the result is propagated backwards in the feedback loop so that the distribution of the generator is as close to that of the real samples as possible while the discriminator is trained to recognize the fake samples. This process continues until the classification probability of an image becomes 0.5, which indicates that the generated image can be regarded as a real one. Therefore, the objective function V(D,G) of GAN can be expressed as a minimax game as follows [10]:

(1) $$\underset{G}{\min}\;\underset{D}{\max}V(D,G)=E_{x\sim p_{data}}[\log D(x)]+E_{z\sim p_{z}}[\log(1-D(G(z)))]$$

where E is the expected value from the data distribution. The last layer of the discriminator is the sigmoid cross entropy loss function denoted as D(x), and it is used to score the probability of distinguishing the real samples which satisfy the condition D(G(z))∈[0,1].

### 2.2. Capsule Network

In the traditional CNN-based architectures, if a certain feature is detected by the convolution kernels, the neurons related to the object will be excited and the output of the object will be generated. However, once some objects in the image are changed from one position to another one such as rotation, translation and scaling, their output based on the original convolution kernels will decrease. Therefore, the change of the direction and spatial hierarchies will affect the feature detectors of CNN. In addition, in order to reduce the size of the feature vectors, the max-pooling strategy is utilized in CNN. In this way, CNN is essentially only interested in the presence of a feature in a certain window, and it generally will not care about the exact location of a feature. If there is a convolution filter for edge detection, then the edges will produce a very high response to this filter, and the max-pooling only retains information related to the edges. Thereby, the “unnecessary” information including the location and spatial relationship between certain features will be thrown away.

The capsule network, as a new type of neural network, will learn robust unsupervised representation of images by utilizing the dynamic routing-by-agreement mechanism between capsules. This network contains the relative location and spatial relationship between objects. Each capsule is a set of neurons, and each neuron represents a certain feature of the object that needs to be detected. The output of the capsule is a vector. The length of the activity vector represents the probability that the entity exists, and the orientation parameters such as rotation and position change are directionally encoded to represent their properties. Once the object has a position variation, the corresponding capsule will output the vectors with the same length but different directions. Thereby, the capsules possess the property of translational equivariance and the whole capsule network will be locally invariant groups of capsules.

**Figure 2:**
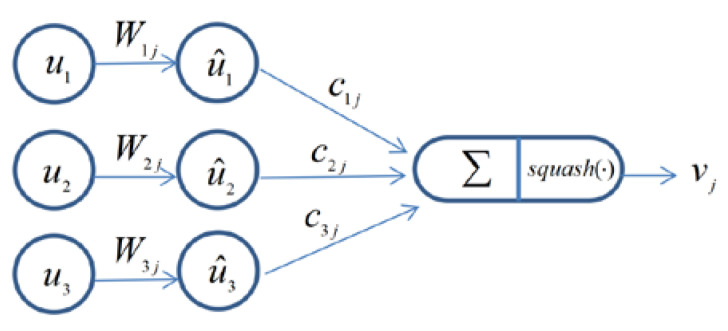
Illustration of the dynamic routing algorithm.

The routing-by-agreement mechanism in the capsule network is an important procedure, which is utilized to replace max-pooling in CNN. Related studies have showed that this type of “routing-by-agreement” is much more effective than the very primitive form of routing implemented by max-pooling, which allows neurons in one layer to ignore all except the most active feature detector in the local pool of the next layer. The details of dynamic routing algorithm are presented in Figure 2. The input s in layer l+1 is calculated by the capsules ${\hat{u}}_{1},{\hat{u}}_{2},{\hat{u}}_{3}$ in layer l as shown in Equation (2) [24]:

(2) $$s_{j}=\sum_{i}c_{ij}{\hat{u}}_{i},\begin{array}{cc} & {\hat{u}}_{i}=W_{ij}u_{i} \end{array}$$

where $u_{i}$ is the output from the capsules in the above layer, W is the corresponding weight of each output which can be regarded as the influencing degree between capsules in successive layers. $c_{ij}$ is the coupling coefficient which is determined by Equation (3) [24]:

(3) $$c_{ij}=\frac{\exp(b_{ij})}{\sum_{k}\exp(b_{ik})}$$

where $b_{ij}$ is given by [24]:

(4) $$b_{ij}\leftarrow b_{ij}+{\hat{u}}_{i}\cdot v_{j}$$

In the process of updating s with back-propagation, the initial W and $b_{ij}$ are set to the random value and 0, respectively. After $c_{ij}$ is calculated, s is produced based on the output $u_{i}$. The non-linear squash function is then used for s to obtain the neuron vector $v_{j}$ of this layer [24]:

(5) $$v_{j}=\frac{\left|\right|s_{j}|{|}^{2}}{1+||s_{j}|{|}^{2}}\frac{s_{j}}{\left||s_{j}|\right|}$$

The first item on the right side of Equation (5) is the scalar of the input vector s for ensuring that the short vectors will shrink to almost zero length and long vectors will shrink to a length slightly less than 1. The second item is the unit vector of s. The squash activation function not only preserves the direction of the input vector, but also compresses the modulus of the input vector to between [0, 1]. Generally, the main characteristic of the capsule network is that the length of the output vector of a capsule is utilized to determine the probability of an object representation, the larger modulus represents the greater probability. By using the obtained parameter $v_{j}$, $b_{ij}$ can be updated based on Equation (4), and $c_{ij}$, s, $v_{j}$ will be updated in turn. Such an update process is the iterative routing-by-agreement mechanism, which is the major difference between the capsule network and CNN.

## 3. Methodology 

**Figure 3:**
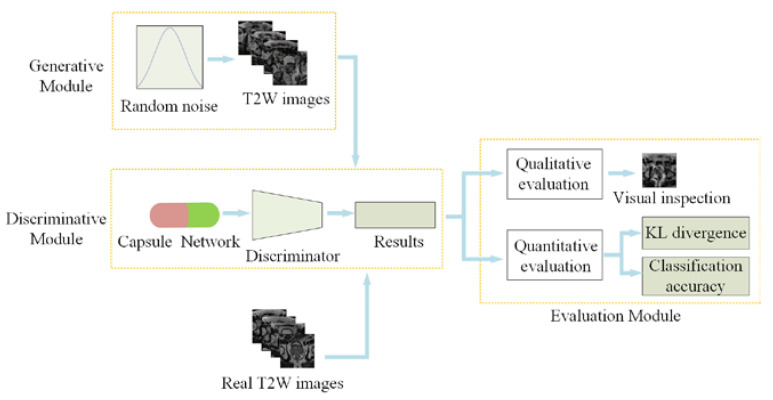
The framework of Capsule Network-Based Generative Adversarial Network (CapGAN)-based MR image generation and evaluation.

In this section, we will present CapGAN-based MR image generation and evaluation. Figure 3 shows the corresponding overall framework. As for CapGAN-based MR image generation, it generally involves two steps: (1) the design of network architecture and (2) model learning from training data. For the design of network architecture, the deep convolutional network architecture is implemented in the generator and the discriminator as the baseline framework of CapGAN by emulating a public DCGAN network. To ensure that the proposed model is suitable for synthesizing medical images, the capsule layers are incorporated into the discriminator of the original DCGAN to replace the conventional convolutions. For the learning of CapGAN, we have adopted the least squares loss for both the generator and the discriminator to improve the stability and generate higher quality images. As for image quality evaluation, we have built a module for qualitative and quantitative evaluation. Qualitative evaluation is conducted through visual inspection while quantitative evaluation is performed in terms of KL divergence and classification accuracy. 

### 3.1. Network Architecture 

**Figure 4:**
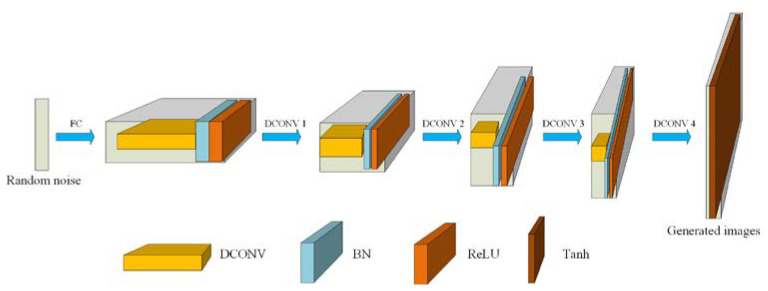
The architecture of the generator of CapGAN.

The generator of our CapGAN incorporates five convolution transpose layers, and its architecture is shown in Figure 4. It takes a uniform 100 dimensional vector as the input, and then the vector is up-sampled to a 4 × 4 × 1024 dimensional vector by utilizing the fully connected (FC) layers of the generator. Starting from the second layer, a successive series of four deconvolution (DCONV) are used to convert this high level representation into a 64 × 64 image. In particular, each convolutional layer in the generator uses batch normalization (BN) to normalize the output of the feature layer. Such processing is beneficial to speed up the network training, improve the network stability and accelerate convergence speed of the learning [27]. Meanwhile, the ReLU activation function is used in all layers except that the Tanh function is used in the last layer. The reason for using the Tanh function is that the last layer needs to output an image, and the pixel value of the image is in the range of [0, 255]. The ReLU function may produce the very large value while the output of the Tanh function in the range of [−1, 1]. The produced value is added by 1 and then multiplied by 127.5. In this way, a pixel value of 0–255 can be obtained.

**Figure 5:**
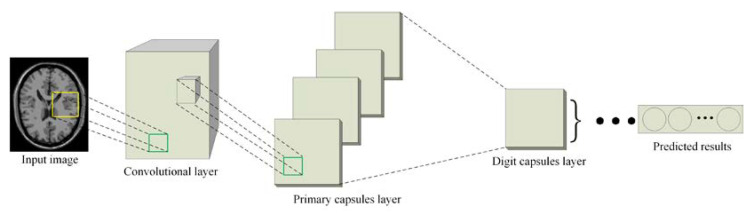
The architecture of the capsule network in the CapGAN.

Since the capsule network can capture the relationship between deep features of different objects in a more robust way, we will introduce it into the discriminator of CapGAN to replace the convolution architecture. The structure of the capsule network is presented in Figure 5. The implementation details of this network are summarized as follows. 

Step 1: Low-level features extraction. For the first layer of the capsule network, the conventional convolution operations are implemented on the input image. Considering that the larger convolution kernel can obtain a larger receptive field in the shallow layer, the size of the convolution kernel is set to 9 × 9. The main function of this step is to detect the local feature information in the image. The capsule is not used because it represents the “current state” of an object, and it is more suitable for the extraction of higher-level features. By comparison, CNN is very effective in representing the low-level features. Therefore, the convolution processing is firstly implemented in the capsule network. The obtained low-level features are then processed by BN and LeakyReLU, where the LeakyReLU is used instead of the ReLU as the activation function because it not only carries the negative information of the feature, but also allows a small negative slope to avoid gradient vanishing. The LeakyReLU function is defined as:

(6) $$y_{i}=\{\begin{array}{c} x_{i}\begin{array}{cc} & if\begin{array}{cc} & x_{i}\geq 0 \end{array} \end{array}\\ \frac{x_{i}}{a_{i}}\begin{array}{cc} & if\begin{array}{cc} & x_{i}<0 \end{array} \end{array} \end{array}$$

where $a_{i}$ is a parameter for controlling the slope of the negative information and it is chosen as 0.2.

Step 2: Translation of the low-level features into the primary capsules. To represent the “current state” of an object, the main capsule architecture starts from this step. The primary capsules include convolution, reshape and squash functions. The convolution is the lowest layer of primary capsule and it has the same number of filters as the previous layer. A reshape function is followed to split all output neurons into the eight-dimensional vector. The features obtained in Step 1 are convoluted with eight-dimensional convolution kernels to generate the main capsule layer in the capsule network. Then, the squash function is utilized to shrink the produced feature maps to the range of [0, 1]. Finally, they are flattened and be treated as the input to a higher-level capsule.

Step 3: Passing values from the primary capsule layer to the digit capsule layer. The capsules obtained in Step 2 are translated to the digit capsule layer based on the dynamic routing algorithm, which is the main difference between the capsule network and the traditional CNN. It should be noted that the original capsule network uses the squash function to shrink long vectors at the end of each dynamic routing, which will produce the artifacts in the generated image. In order to more accurately retain the features produced from training, we will replace all squash functions with LeakyReLU to reduce the artifacts and improve the quality of the generated images.

Step 4: Using the final outputs from digit capsule layer as the prediction vector. By calculating the L2 norm (i.e., the length) of the vectors produced in Step 3, we can obtain the final prediction result of the input image.

### 3.2. The Objective Function of CapGAN

In the original capsule network, the margin loss is used as the loss function, and it is defined as [24]:

(7) $$L_{k}=T_{k}\max{\left(0,m^{+}-||v_{k}||\right)}^{2}+\lambda (1-T_{k})\max{\left(0,||v_{k}||-m^{-}\right)}^{2}$$

where $T_{k}$ is the target label which represents the probability that a capsule’s entity exists and will be equal to 1 if the object of class k exists in the image and 0 otherwise. $m^{-}$ and $m^{+}$ are hyper-parameters and they are set to 0.1 and 0.9, respectively. λ is the ratio coefficient between $m^{-}$ and $m^{+}$ and it is set to 0.5 through hyper-parameter search. $v_{k}$ is the final output capsule vector. The total loss function is the sum of all $L_{k}$.

In our study, the squash function of capsule network is replaced with the LeakyReLU activation function to reduce the artifacts in the generated image. At the same time, we hope that the proposed CapGAN can avoid the multiple-class prediction. Therefore, the margin loss is unsuitable for detecting if an image is real or fake. Another commonly used strategy is to adopt the sigmoid cross entropy loss function. However, it may lead to the unstable training because of the vanishing gradient problem [21]. When the parameters of the generator are fixed, Arjovsky et al. [19] have proved that the optimal discriminator in this case can be expressed as:

(8) $$D_{G}^{*}(x)=\frac{p_{data}(x)}{p_{data}(x)+p_{g}(x)}$$

where $p_{g}\left(x\right)$ is the distribution of samples produced by the generator.

While the optimal discriminator is derived, the optimal objective of the generator is [19]:

(9) $$\begin{array}{ll} C(G) & =\underset{D}{\max}V(G,D)\\ & =E_{x\sim p_{data}}[\log D_{G}^{*}(x)]+E_{z\sim p_{z}}[\log(1-D_{G}^{*}(G(z)))]\\ & =E_{x\sim p_{data}}[\log D_{G}^{*}(x)]+E_{x\sim p_{g}}[\log(1-D_{G}^{*}(x))]\\ & =E_{x\sim p_{data}}[\log\frac{p_{data}(x)}{p_{data}(x)+p_{g}(x)}]+E_{x\sim p_{g}}[\log\frac{p_{g}(x)}{p_{data}(x)+p_{g}(x)}] \end{array}$$

Equation (9) can be represented succinctly by Jensen-Shannon divergence (JSD) [20]:

(10) $$C(G)=-\log(4)+2\cdot JSD(p_{data}||p_{g})$$

As Equation (10) shows, when the discriminator is the optimal, the training goal of GAN is to minimize the JSD between the real data distribution and the generated one. Generally speaking, minimizing JSD may lead to the vanishing gradient problem. Besides, if the discriminator is trained very well, the generator will not be able to learn the gradient effectively. Conversely, if the discriminator is trained very weakly so that the discrimination ability is not significant, this will lead to the situation that the generator cannot learn the gradients effectively. Therefore, the objective function will cause the instability of learning in GAN, and it needs really careful coordination for the training degree of the generator and the discriminator during the learning process. This is the main reason why the training of GAN is a difficult issue in practice.

In order to develop a more suitable technique for measuring the distance between the real data distribution and the generated data distribution, the least squares loss function as the objective function is suggested for training GAN [21]. By using the least squares loss, the data that are far from its decision boundary will be penalized proportionally based on the distance to this boundary. Therefore, the only way to minimize the loss function of the discriminator is to force the generator to generate the samples as close to the decision boundary as possible. Through such an optimization process, the generator can gradually learn to match the real data. Compared with the sigmoid cross-entropy loss function, the least squares loss function performs more stably and can generate higher quality images. In the proposed CapGAN model, we will use the least squares loss function as the objective function for both the discriminator and generator. Here, the objective functions are defined as [21]:

(11) $$\underset{D}{\min}V_{CapGAN}(D)=\frac{1}{2}E_{x\sim p_{data}}[{\left(D(x)-1\right)}^{2}]+\frac{1}{2}E_{z\sim p_{z}}[D{\left(G(z)\right)}^{2}]$$

(12) $$\underset{G}{\min}V_{CapGAN}(G)=\frac{1}{2}E_{z\sim p_{z}}[{\left(D(G(z))-1\right)}^{2}]$$

where $V_{CapGAN}\left(D\right)$ and $V_{CapGAN}\left(G\right)$ are the loss function of the discriminator and the generator, respectively. z is the noise vector obeying Gaussian distribution, $E_{x\sim p_{data}}$ and $E_{z\sim p_{z}}$ denote the same values as those in Equation (1).

### 3.3. The Evaluation Metrics 

The evaluation of the quality of images produced by GAN remains a challenging task. Several methods have been introduced to address this issue in recent years. Goodfellow et al. [10] have proposed to compare the generated samples and its nearest neighbors in the data to estimate image quality. Inception score (IS) proposed by Salimans et al. [28] has been suggested as the ad hoc quantitative measure correlated with the visual quality of generated images. It is calculated by the KL divergence between the (logit) response produced by this image and the marginal distribution based on the Inception network [29] trained on ImageNet. As a result, IS is limited to quantify the diversity of the generated samples because the samples are not compared with the target distribution. To overcome the drawback of IS, Fréchet Inception distance (FID) has been presented by Heusel et al. [30] to further evaluate GAN. As a widely used quantitative metric, FID compares Inception activation between the real samples and the generated ones. However, this comparison approximates the activation between real and generated samples as Gaussian distribution by calculating their mean and covariance, which will result in the disadvantage that they are too crude to capture subtle details [31]. Both these measures rely on the Inception network pre-trained on ImageNet, which is far from ideal for other datasets such as face and biomedical images.

**Figure 6:**
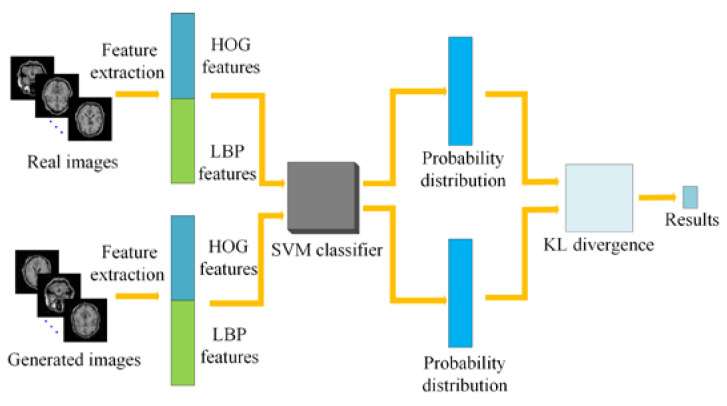
A flowchart for evaluating the difference between real and generated MR images.

As there are insufficient medical images in ImageNet, it is unreliable to evaluate GANs by computing IS and FID based on the generated medical samples. Motivated by the principles of IS and FID, we will try to train a simple classifier for evaluating the generated medical images. Here, the binary classification problem of medical images is considered. As support vector machine (SVM) has been successfully applied in this field [32], we will first train a SVM model on MR images, and then the real images and the generated images were input into the trained model. Finally, the KL divergence between them is calculated according to the probability distribution of the two types of images based on the pre-trained classifier. The result will demonstrate the similarity between the generated images and the real ones. The proposed evaluation flowchart is shown in Figure 6. The implementation details are described as follows:

Step 1: The histograms of oriented gradient (HOG) and local binary pattern (LBP) features are extracted from MR images in the training set, and then they are cascaded and sent into the SVM model for training a SVM classifier. The main reason for utilizing the HOG and LBP features is that HOG features can represent the appearance and shape information of local objects in MR images [33] while LBP features can represent local texture information in MR images and they are invariant to rotation and gray scale [34].

Step 2: The same number of real MR images and fake MR images generated by GAN are selected to extract HOG and LBP features, respectively. Then, they are cascaded together.

Step 3: The two classes of features obtained from Step 2 are sent to the SVM classifier trained in Step 1 to produce the corresponding probability distribution.

Step 4: According to the probability distribution of the two classes of images obtained in Step 3, the KL divergence between them can be calculated by the Equation (13) [20] to determine the difference between the generated image and the real image.

(13) $$D_{KL}(P||Q)=\int_{x}P(x)*[\log(\frac{P(i)}{Q(i)})]dx$$

where $D_{KL}$ is the divergence between two distributions P,Q∈Prob(X). 

## 4. Experimental Results and Discussion

To evaluate the effectiveness of CapGAN in generating realistic MR images, several experiments have been performed on different datasets including the well-known BrainWeb phantom [35] and the real prostate MRI dataset [36,37,38]. The evaluation of CapGAN is made from two aspects. On one hand, the performance of the CapGAN model is compared with that of two related GANs including DCGAN and LSGAN qualitatively and quantitatively. Specifically, the qualitative analyses are conducted through two radiologists with 15 years of clinical experience while the quantitative results are obtained by the self-designed evaluation method presented in the previous section. On the other hand, to further demonstrate the superiority of CapGAN to other compared models for image classification, a prostate MR image classification method is adopted and it is trained on the images augmented by the traditional methods and three GANs to evaluate the classification performance. In all experiments in this study, since DCGAN, LSGAN and CapGAN models are well-suited for parallel computation on GPU, we use the NVIDIA cuDNN-7.0 deep learning library to accelerate the GPU computation of GANs. These models are run on a computer with the following environment including GPU (NVIDIA GeForce GTX 1060), CPU (Intel Core i5-6300), and RAM (24G DDR4). All training processes are implemented with Keras on TensorFlow platform, and each dataset is trained for 50,000 epochs. As for the learning rate, it is initialized as 0.0002 and is dynamically adjusted by Adam optimizer. For other training hyper-parameters, the suggested values by authors in the related references are firstly used as the initial values, and then they are further fine-tuned using a grid search from the narrow hyper-parameter ranges to ensure the best performance. 

### 4.1. Comparison of GANs in Terms of Generated Image Quality 

**Figure 7:**
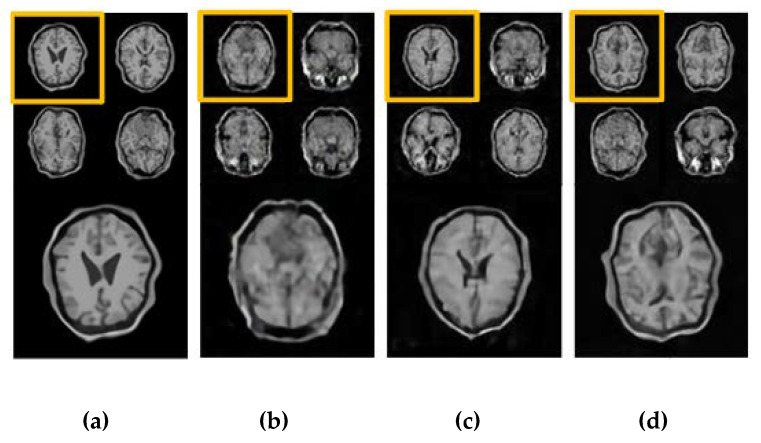
The generated brain T1W images and the zoomed results of images marked with orange color for different GAN-based models: (**a**) the real images, (**b**) Deep Convolutional GAN (DCGAN), (**c**) Least Squares GAN (LSGAN), and (**d**) CapGAN.

Testing has been done on the simulated brain MR images and the real prostate MR images. Here, the simulated T1W MR images are taken from the BrainWeb dataset available at http://brainweb.bic.mni.mcgill.ca/brainweb [35]. The total number of original images is 1400, and each image of size 64 × 64 has been processed with 10-fold rigid and non-rigid deformation to expand the dataset to 14,000 training images for this task. Figure 7 shows the results between the real images and the images generated by the three GAN models including DCGAN, LSGAN and CapGAN. It can be seen from Figure 7 that the images generated by DCGAN are unsatisfactory because there exists the loss of structural information, edge incompleteness and blurring of the internal texture details. The images generated by LSGAN are obviously better than those generated by DCGAN in that the former have relatively clear edges and some internal structure details can be observed, but some regions are still blurred. In comparison, the generated images by CapGAN have better quality and they are closer to the real images in that they have clear and complete brain edges, and internal texture details as well as small structures are also be clearly seen. Furthermore, these images are analyzed by two radiologists with 15 years of clinical experience, their viewpoints are that the images generated by CapGAN looks more realistic, the boundary between white matter and gray matter is more distinct, and the sulci and gyri can be more clearly observed.

The real prostate MR images are acquired from the open MRI database released for PROSTATEx Challenge as part of the 2017 SPIE Medical Imaging Symposium [36,37,38]. The entire dataset consists of 325 mp-MRI cases, in which each case contains T2-weighted (T2W) images (transaxial, sagittal and coronal), ADC images, DWI images, Proton Density weighted (PD) images and DCE images. In this experiment, the T2W, ADC and DCE images have been chosen for testing the performance of three GANs. The reason of using the three modality images can be explained in this way. T2W shows more detailed anatomical structures of the prostate [39]. DWI can evaluate degree of invasion in quality and quantity, but they are commonly converted to ADC maps [40,41], which show better representation of lesions than DWI images [39]. As benign prostatic hyperplasia nodules may be highly vascular on DCE images, DCE may increase the likelihood that the finding corresponds to a clinically significant cancer [4].

Considering that the number of images is quite scarce, there is a high probability that this will lead to overfitting. To address the problem, we have applied the feature-based transfer learning method to prevent overfitting during the training process. The detailed implementation is given as follows:

Step 1: For each modality image, we have sliced all sequence data into single images. In this way, we have obtained about 6000 images.

Step 2: The patches are selected by cropping the image obtained in Step 1 to a 35 × 35 region of interest (ROI) surrounding the lesion center. The reason for such processing is that the large MR image is not easy for analysis due to the included great amount of information, and the information far away from the lesion center has little impact on the classification of prostate cancer lesions. 

Step 3: The patches produced in Step 2 is augmented by applying shifting, cropping, rotation and adding noise for improving GAN training. The augmented dataset contains 24,000 images.

Step 4: The pre-trained networks can be obtained by utilizing these 24,000 images as training set to train the three GANs, respectively.

Step 5: The original 325 marked lesion images are cropped as done in Step 2, and then they are used as training samples and input into GANs to generate images, in which the weights of pre-trained networks in Step 4 are applied as the initial parameters. Since the same modality images have relevant features, transfer learning can speed up the training process and avoid overfitting during the learning process effectively [42]. Accordingly, the quality of generated images by transfer learning is better than that by the regular learning.

**Figure 8:**
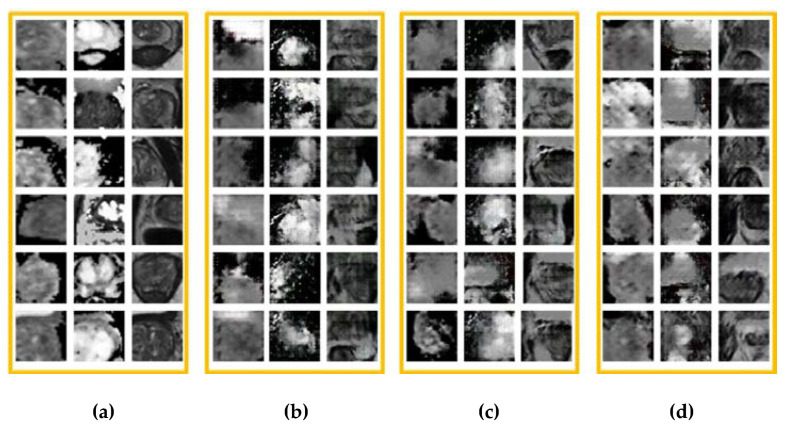
Comparison of real and generated MR images, where the first, second and third columns in each orange box are ADC images, DCE images and T2W images, respectively: (**a**) the real images, (**b**–**d**) are the generated images using the DCGAN, LSGAN, and CapGAN models, respectively.

Figure 8 shows some different modalities of real MR images and generated MR images. Compared with real images, the images generated by DCGAN and LSGAN seem to be blurry, the structure is incomplete and there is still some noise in these images. In particular, the loss of image details exists and many strip artifacts can be observed in the T2W images generated by DCGAN. For the images generated by CapGAN, the clearer and complete boundaries can be observed in the DCE and T2W images, and these images look realistic and have not obvious artifacts. The above comparison indeed demonstrates the superiority of CapGAN over the compared GANs because the images it generates are closer to the real ones. In addition, it should be noted that there still exists slight differences between the generated ADC and DCE images by CapGAN and the real images. The main reason is that the resolution of the two modalities of images is relatively low and our training samples are not sufficient although the transfer learning method is used. 

Furthermore, we have calculated the KL divergence between the original simulated T1W images and the generated images and between the real prostate MR images and the generated images based on Equation (13), respectively. The smallest value for each modality image is represented in bold. It can be seen from Table 1 that CapGAN achieves the minimum KL divergence value for the simulated images, which demonstrates that the images generated by CapGAN are closer to the real ones. For the real prostate data, CapGAN provides the smallest KL divergence for DCE and T2W images among all evaluated methods although it provides higher value for the ADC images than LSGAN. The above quantitative comparison is consistent with the previous visual appreciation.

### 4.2. Comparison of GANs in Terms of Classification Accuracy 

To further demonstrate the superiority of CapGAN in generating images for classification task, we have performed the binary classification experiment on the real prostate cancer dataset via the traditional augmentation (TraAug), DCGAN-based augmentation (DCGANAug), LSGAN-based augmentation (LSGANAug), and CapGAN-based augmentation (CapGANAug) methods. Recent studies [43] have demonstrated that the early layers of deep learning models can be replaced with predefined representations to reduce and parametrize variability while retaining the discriminative information. Inspired by this motivation, we have proposed to combine feature representation with the micro networks for prostate MR image classification. The proposed model combines Laplacian eigenmaps network (LENet) [25] with a light-weight network-in-network (NIN) [26] to address the overfitting problem. In the hybrid model, LENet is firstly used to extract the intrinsic features from the original MR images. As described in [25], our proposed LENet includes two convolutional filter bank layers based on Laplacian Eigenmaps algorithm. The obtained features are then input into NIN [26] for deep feature learning. We will use the combined model LENet-NIN to test the performance of the traditional augmentation method and GANs based method. 

In this experiment, a total of 325 lesion images (75 malignant lesions and 250 benign ones) are marked on these images. We have used 80% lesion images as the training set which includes 60 malignant lesions and 200 benign ones, and used the remaining 20% lesion images as the testing set which includes 15 malignant lesions and 50 benign ones. The assignment of each lesion to the training set or testing set will be random. Since the number of training samples is small, data augmentation is necessary for increasing the amount of data. We have used the TraAug and GANs-based methods to increase the training images by 20 times. Here, the TraAug method means the operation of rotation, translation and scaling on the images. Totally, 5460 images have been obtained and they will be input into the LENet-NIN for training. Considering that the distribution of samples is extremely imbalanced (the ratio of the number of positive samples to that of negative samples reaches about 1:3), it is not enough only to use accuracy as the evaluation metric. Here, accuracy is defined as the ratio of the number of correctly predicted samples to that of all samples. Therefore, we have also used the receiver operating characteristic (ROC) curve as the metric for analyzing the classification performance [44]. 

**Figure 9:**
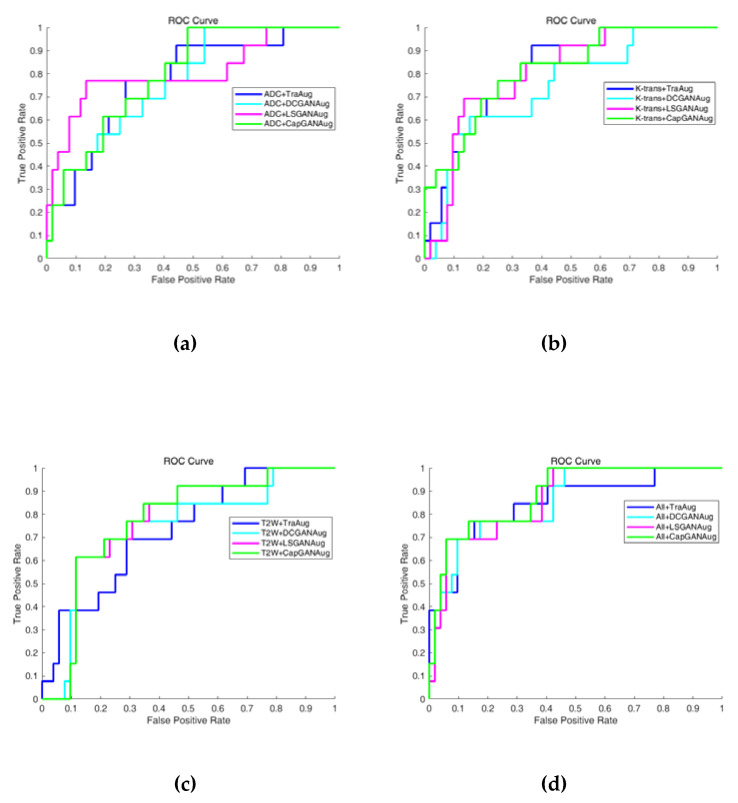
The ROC curves of different modalities of MR images by using the traditional data augmentation strategy and three GAN-based augmentation methods: (**a**) ADC images, (**b**) K-trans images, (**c**) T2W images, and (**d**) ADC+K-trans+T2W images.

Figure 9 shows the classification results of three single-modality images and multiparameter MR images by using the traditional-based augmentation and GANs-based augmentation. The blue, cyan, magenta and green ROC curves are produced by using TraAug, DCGANAug, LSGANAug and CapGANAug methods, respectively. It can be seen that the green ROC curves are closer to the upper left, and the larger area under the curve (AUC) can be achieved by CapGANAug for K-trans, T2W and ADC+K-trans+T2W images. The above observation demonstrates that the proposed CapGANAug method yields the best classification results for four types of images except the ADC images among all evaluated methods. Therefore, the images generated by CapGAN generally can facilitate improving the classification performance very effectively.

In addition, the quantitative results in terms of classification accuracy and AUC are listed in Table 2. Obviously, the CapGAN method achieves higher accuracy and AUC value for single-modality MR image and multiparameter MR images. These results show that the CapGAN method is more effective than the traditional method and the compared GANs in generating the training samples for the presented deep learning-based classification method.

### 4.3. Comparison of GANs in Terms of Complexity

The baseline framework of the proposed model is designed by emulating a public DCGAN network. The difference of time and space complexity among CapGAN, DCGAN and LSGAN are mainly determined by the difference of CNN and capsule network. Although the capsule network has higher complexity than CNN, they are only incorporated into the discriminator to replace CNN. Besides, the dataset size in this study is small. Therefore, there are not great differences of time complexity and space complexity among three GANs. Table 3 lists the number of parameters and training time of the proposed CapGAN and the most competitive LSGAN operating on the brain T1W images. Table 3 shows that the training time of CapGAN is only 5.7 min more than that of LSGAN although it involves more parameters. 

## 5. Conclusions 

In this study, a new GAN architecture has been designed for MR image synthesis. Distinctively, the proposed method has introduced the capsule network into the discriminator of the proposed GAN model to replace the traditional convolution architecture. Furthermore, we have used the least squares loss instead of the cross-entropy loss to improve the stability of the proposed GAN. The introduction of the capsule network and the least squares loss indeed ensures the quality and the diversity of generated images. To evaluate the performance of the proposed GAN in image generation, a new KL divergence-based appreciation method is presented. Both qualitative and quantitative results on the simulated and real MR dataset have demonstrated the superiority of the proposed model over the compared models in generating realistic and useful images for such applications as image classification. Indeed, the proposed model has shown the potential to address the problem of inadequate samples and diversity of medical image in deep learning. In future, the new GAN will be designed for synthesizing the low-resolution images.

## Figures and Tables

**Table 1:** The results of Kullback–Leibler (KL) divergence on the MR dataset for the DCGAN, LSGAN and CapGAN models.

Images	Modality	DCGAN	LSGAN	CapGAN
Simulated Images	T1	1.1687	1.1315	**1.0961**
Real Images	ADC	1.1591	**0.7494**	0.8086
	DCE	1.4858	1.0317	**1.0086**
	T2W	0.8213	0.8072	**0.7333**

**Table 2:** The accuracy and area under the curve (AUC) of four data augmentation methods on different modalities of MR images.

Metrics	Data Augmentation	ADC	K-Trans	T2W	All
Accuracy	TraAug	0.754	0.789	0.754	0.815
	DCGANAug	0.785	0.769	0.754	0.831
	LSGANAug	**0.831**	0.789	0.769	0.862
	CapGANAug	0.815	**0.800**	**0.785**	**0.892**
AUC	TraAug	0.774	0.812	0.731	0.851
	DCGANAug	0.769	0.784	0.744	0.855
	LSGANAug	**0.806**	0.805	0.769	0.865
	CapGANAug	0.796	**0.817**	**0.772**	**0.885**

**Table 3:** The comparison of parameters and training time for CapGAN and LSGAN on the brain T1W images.

Models	Total Parameters	Training Time (s)
LSGAN	≈7.2 × 10^6^	9923
CapGAN	≈9.1 × 10^6^	10,265
